# Human Pharmacokinetics of High Dose Oral Curcumin and Its Effect on Heme Oxygenase-1 Expression in Healthy Male Subjects

**DOI:** 10.1155/2014/458592

**Published:** 2014-01-29

**Authors:** Uros Klickovic, Daniel Doberer, Ghazaleh Gouya, Stefan Aschauer, Stefan Weisshaar, Angela Storka, Martin Bilban, Michael Wolzt

**Affiliations:** ^1^Department of Clinical Pharmacology, Medical University of Vienna, Waehringer Guertel 18-20, 1090 Vienna, Austria; ^2^Department of Pulmonary Medicine, Wilhelminenspital Vienna, 1160 Vienna, Austria; ^3^Clinical Institute of Medical and Chemical Laboratory Diagnostics, Medical University of Vienna, 1090 Vienna, Austria

## Abstract

*Purpose*. Heme oxygenase-1 (HO-1) has been proposed to exert pharmacological benefits by its antioxidative and anti-inflammatory effects. HO-1 expression may be affected by the GT length polymorphism in the promoter region of the HO-1 gene. We investigated the inducibility of HO-1 by orally administered curcumin in healthy male subjects and its correlation with the GT length polymorphism. *Methods. *In an open label uncontrolled phase-1 pilot study, ten male subjects received 12 g of oral curcumin. To investigate the effects of the GT length polymorphism on the inducibility of HO-1, five subjects with homozygous short and five with homozygous long GT genotypes were studied. Plasma concentrations of curcumin, bilirubin, HO-1 mRNA, and protein expression in peripheral blood mononuclear cells (PBMCs) were analyzed over 48 hours. *Results.* At a detection limit of 1 *µ*g/mL curcumin could not be detected in plasma of any subject. Compared to baseline, HO-1 mRNA and protein levels were not induced in PBMCs at any time point up to 48 hours. There was no correlation between any of the parameters and GT length polymorphism. *Conclusions*. Oral curcumin administration has low bioavailability and does not induce HO-1 on mRNA or protein level in PBMCs.

## 1. Background Information

### 1.1. Curcumin and Heme Oxygenase

Curcumin, a bioactive component of turmeric, has been proposed to modulate multiple cell signaling pathways and to interact with numerous molecular targets, including cell cycle, apoptosis, proliferation, angiogenesis, and inflammation [1]. Beside its beneficial effect on cancer cells [2], orally ingested curcumin improved glucose metabolism in type 2 diabetes animal models [3]. Curcumin is also suggested to be a stimulant of heme oxygenase-1 (HO-1). This has been shown *in vitro* in human breast cell lines [4] and in human hepatocytes. The strong therapeutic potential of curcumin analogues for treating diabetic diseases and their ability to induce HO-1 expression has also been discussed in the recent published article by Son et al. [5].

HO-1 is the rate-limiting enzyme that catalyzes the degradation of heme b (Fe-protoporphyrin-IX) into biliverdin (which is rapidly converted to bilirubin), carbon monoxide (CO), and free iron (Fe^2+^). In humans two genetically distinct isozymes of HO have been characterized: a constitutively expressed form HO-2 and an inducible form HO-1 [6]. HO-1 is a member of the heat-shock protein family (HSP 32) being expressed in endothelial, epithelial, and smooth muscle cells [7]. It serves as a protective enzyme due to its anti-inflammatory, antioxidant, antiapoptotic, and antiproliferative mechanisms of actions [8]. There is a GT length polymorphism (GT)n dinucleotide repeat polymorphism in the proximal promoter region of the HO-1 gene [9]. This (GT)n repeat is highly polymorphic and modulates gene transcription by means of oxidative challenge [10]. *In vitro* studies evidenced that a longer (GT)n repeat corresponds to lower transcriptional activity of the HO-1 promoter region [11, 12] and is associated with a susceptibility to large number of diseases [13], including the coronary artery disease in type 2 diabetic patients [14, 15]. Oral administration of curcumin to patients after cadaveric renal transplantation led to an increase of HO-1 protein levels in urinary epithelial cells and improved renal function [16]. The molecular steps and signal transduction pathways underlying the HO-1 upregulation in general, and by curcumin in particular, remain largely undefined. PI3K and p38^MAPK^ pathways under the control of the transcription factor NF-E2 related factor 2 (Nrf2) and NF-*κ*B might play a substantial role in the HO-1 induction [2, 17, 18].

However, bioavailability of curcumin after oral administration is low [19] due to extensive metabolic breakdown in the gastrointestinal tract [20]. To date, there are no sufficient studies available that have assessed the pharmacokinetics of a single high dose oral curcumin and its association with HO-1 expression. We therefore investigated the pharmacokinetics of a single high dose oral curcumin and its ability to induce HO-1 expression in humans among different human specific (GT)n polymorphisms.

## 2. Methods

### 2.1. Study Participants

**Figure 1:**
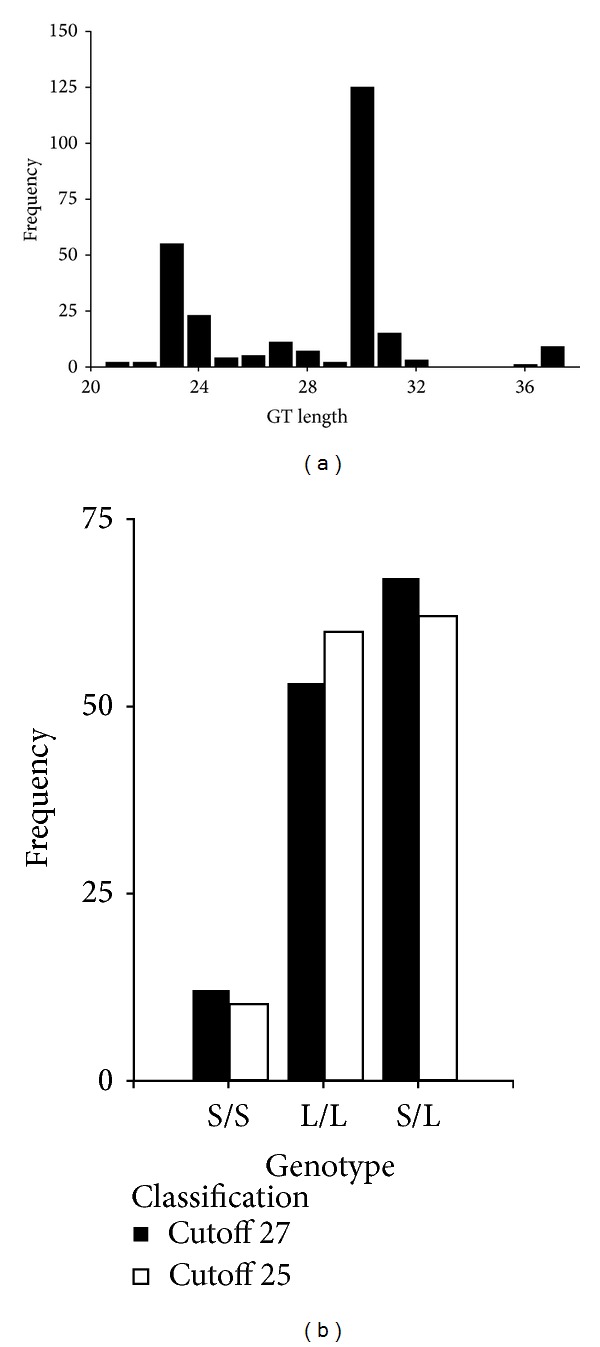
HO-1 genotype assessment. (a) Allele distribution of the heme oxygenase gene from 132 subjects. The GT length is shown on the *x*-axis and allele frequency on the *y*-axis. (b) Prevalence of the GT length genotypes for the cutoff 27 (S: < 27; L: ≥ 27).

**Figure 2:**
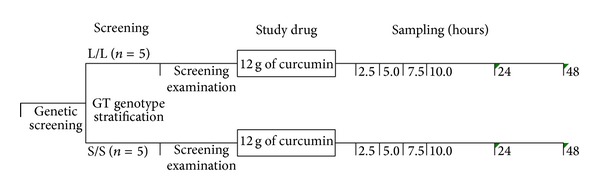
Schematic study design. Five subjects with a homozygous short GT genotype (S/S; age: 29 ± 4; BMI: 23.7 ± 2.0 kg/m²) and five with a homozygous long GT genotype (L/L; age: 27 ± 4; BMI: 23.7 ± 1.8 kg/m²) were included and exposed to oral curcumin.

132 healthy white European male subjects aged between 18 and 45 years (inclusive) were screened for the (GT)n length polymorphism in the promoter region of the HO-1 gene (Figure 1). Subjects were stratified in two groups according to their HO-1 genotype, which may affect transcription of the HO-1 gene after oral curcumin administration. Five subjects with a homozygous short GT genotype (S/S; age: 29 ± 4; BMI: 23.7 ± 2.0 kg/m²) and five with a homozygous long GT genotype (L/L; age: 27 ± 4; BMI: 23.7 ± 1.8 kg/m²) were included in this study and exposed to oral curcumin (Figure 2). The study protocol was approved by the Ethics Committee of the Medical University of Vienna, and written informed consent was obtained from all participants before study entry. The study is registered at ClinicalTrials.gov (NCT 00895167).

### 2.2. Study Design

In this open label, uncontrolled phase-1 pilot study, each participant passed a screening examination that included medical history, a physical examination, vital sign measurement, a 12-lead electrocardiogram, laboratory tests, drug screening, and test-strip urinalysis between 2 and 14 days before the first drug administration. 12 g of oral curcumin (12 capsules containing a powder of Curcumin C3 Complex) was administered after an overnight fast. Curcumin and total bilirubin plasma levels, HO-1 mRNA, and protein expression in peripheral blood mononuclear cells (PBMCs) were analyzed before and at 2.5, 5, 7.5, 10, 24, and 48 hours after the study drug administration. Laboratory safety tests including chemistry, hematology, coagulation, and urine chemistry were performed at screening and at the end-of-study visit (EOS). Vital sign measurement and a 12-lead electrocardiogram were recorded during the investigational period at −0.5 and 7.5 hours and at EOS. Adverse events were recorded throughout the study.

### 2.3. Study Drug

Curcumin C3 Complex (Sabinsa Corporation, NJ, USA) is known to be well tolerated when taken at high doses of 12 g/day [20, 21]. The study drug consists of 73–78% curcumin I (curcumin; molecular formula: C_21_H_20_O_6_; molecular weight: 368.380 g/mol); 18–22% curcumin II (demethoxycurcumin; molecular formula: C_20_H_18_O_5_; molecular weight: 338.354 g/mol); and 2–5% curcumin III (bisdemethoxycurcumin; molecular formula: C_19_H_16_O_4_; molecular weight: 308.328 g/mol). In order to increase bioavailability of this complex and to inhibit rapid drug decomposition, the study drug contains 5 mg Bioperine, which is a standardized extract from the fruits *Piper nigrum* L and *Piper longum* L, containing 95% of piperine [22].

## 3. Laboratory Assessment

### 3.1. Plasma Curcumin Measurement

Plasma curcumin was analyzed by reversed-phase high-performance liquid chromatography (RP-HPLC) method using a Hitachi LaChrom Elite HPLC with L-2400 UV detector (Hitachi HTA, Life Sciences Division, CA, USA), and Waters *μ*Bondapak C_18_ column. Detection wave lengths were 428 and 300 nm for curcumin and 4-hydoxybenzophenone (as internal standard, IS), respectively. The internal standard (IS) was determined with 50 ng/mL of curcumin. The lower limit of quantification was 1 ng/mL.

### 3.2. HO-1 Genotype Assessment

Genomic DNA was isolated from whole blood using standard techniques. Polymerase chain reaction amplifications of the HO-1 (GT)n repeat length polymorphism were performed as described in [9, 10]. We divided allelic repeats into two subclasses following a classification based on transfection studies with low and high GT repeats [6, 9, 10, 14, 15]: short repeats with <27 (GT)n were designated as allele class S (short) and longer repeats with ≥27 (GT)n as allele class L (long).

### 3.3. HO-1 mRNA Expression in PBMCs

PBMCs were isolated from EDTA blood with Ficoll-Plaque (Amersham Biosciences, UK) prefilled tubes (Leucosep, Greiner Bio-One, Austria). Cell pellets for HO-1 mRNA analysis were treated with lysis buffer (Buffer RLT, Qiagen Sciences, MD, USA). First-strand cDNAs were synthesized from approximately 1 *μ*g of total RNA with the use of MLV reverse transcriptase and random hexamer primers according to the manufacturer instructions (RT-PCR Core Kit; Takara Bio). For quantitative real-time polymerase chain reaction (RT-PCR), sense and antisense primers (Invitrogen, Paisley, Scotland) and fluorogenic probes (Eurogentec, Herstal, Belgium) for HO-1 and 18S were used. Results are expressed as the target/reference ratio. The difference between the HO-1 mRNA levels of curcumin and vehicle-treated cells was considered as the cell expression of HO-1 mRNA and is expressed as ΔHO-1 mRNA.

### 3.4. HO-1 Protein Levels in PBMCs

HO-1 protein levels in PBMCs were detected using Western blot analysis. The dry cells were lysed with a lysis buffer containing protease inhibitors. After centrifugation the resulting supernatant was used for protein concentration measurements using a bicinchoninic acid protein assay kit (Thermo Scientific). 30 *μ*L of a solution containing SDS loading buffer and mercaptoethanol were combined with 50 *μ*g of sample protein and lysis buffer up to 100 *μ*L. Samples were heated at 93°C and separated by polyacrylamide gel electrophoresis (PAGE) at 120 V. They were transferred to polyvinylidene fluoride (PVDF) blot membrane (20 V for 1.5 hrs) using the semidry method. The membranes were blocked in 5% milk for 1 hour, washed three times with TBST (tris-buffered saline including 0.05% Tween-20), incubated with the primary HO-1 antibody over night at 4°C, and washed and incubated with the second antibody for 1 hr. The membranes were incubated in substrate solution and imaged using clear X-ray films. The antibody staining and development procedure was repeated again using an anti-*β*-actin antibody for normalisation of the results.

### 3.5. Safety Laboratory Parameters

Complete blood counts as well as blood, dialysate, and urine chemistry were performed by standard procedures in an ISO 9001: 2000 certified laboratory.

## 4. Statistical Methodology and Analysis

For the statistical analyses, SPSS Statistics 18.0 (SPSS Inc., Chicago, IL, USA) was used. Nonparametric statistics were applied for comparison between groups and within groups. Differences between baseline characteristics were assessed by the Wilcoxon test. The Mann-Whitney-*U* test was performed to assess a possible interaction (effect modification) between genetics and treatment. All *P* values are results of two-sided tests, and *P* values <**  **0.05 are considered statistically significant. Since the study has an exploratory character, no adjustment for multiple testing was performed.

## 5. Results

### 5.1. HO-1 Genotype Characteristics

A total of 132 subjects were screened for the GT length polymorphism in the HO-1 promoter region. The (GT)n repeats ranged between 21 and 37, with 23 and 30 being the most common alleles (Figure 1). Using the cutoff of 27 repeats, the prevalences of the genotypes for homozygous S/S and L/L and heterozygous S/L were 9.1%, 40.2%, and 50.8%, respectively [23].

### 5.2. Baseline Characteristics

Five subjects with a homozygous short (S/S) GT genotype and five with a homozygous long (L/L) GT genotype of the HO-1 length polymorphisms were investigated in our pilot study. The demographic data and relevant baseline laboratory values are summarized in Table 1.

### 5.3. Curcumin Plasma Levels

Curcumin was not detectable before or after oral administration of study drug at any timepoint. RP-HPLC (detection level: 1 ng/mL) did not detect any quantified curcumin plasma levels at any timepoint.

### 5.4. Bilirubin Plasma Levels

Lower levels of conjugated bilirubin were determined in the S/S group (0.15 mg/dL) compared with the L/L group (0.20 mg/dL; *P* = 0.015) at predose. No difference in ΔAUC_48 h_ of mean bilirubin (total fraction and subfractions) could be observed after oral curcumin administration compared with the individual baseline levels of the study participants. Comparing the two predefined genotype groups, no significant difference could be detected for both, total fractions and subfractions of plasma bilirubin.

### 5.5. HO-1 mRNA

**Figure 3:**
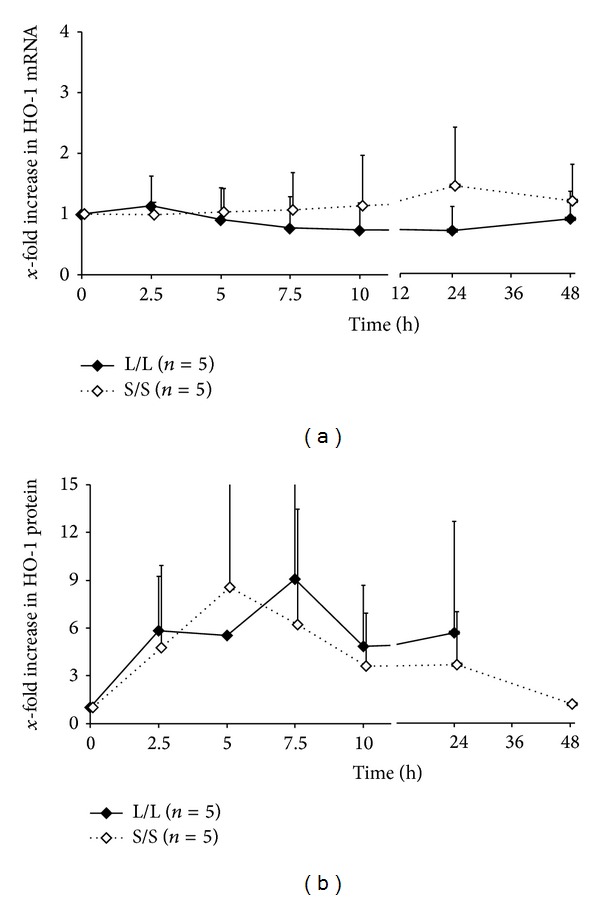
Expression after treatment with 12 g of oral curcumin. (a) HO-1 mRNA and (b) HO-1 protein level.

HO-1 mRNA baseline concentrations of both genotype groups are presented in Table 1. No change in the area under curve over 48 h (ΔAUC_48 h_) of mRNA concentrations from the individual baseline level was observed (*P* = 0.878, Figure 3(a)).

### 5.6. HO-1 Protein Levels

HO-1 protein baseline levels are presented in Table 1. There was no significant difference in the maximal concentration (*C*_max⁡_  L/L : 10.71 versus *C*_max⁡_  S/S : 8.56, *P* = 0.169) or the time to *C*_max⁡_ between the L/L group (*t*_max⁡_  L/L : 5.01) compared with the S/S group (*t*_max⁡_ : 6.52). The area under curve ΔAUC_48 h_ of HO-1 protein did not differ between the groups. For the HO-1 protein, no genetic effects could be detected in the area under curve ΔAUC_48 h_ (*P* = 0.459) and maximal concentration *C*_max⁡_ (*P* = 0.169) comparing both genotypes.

### 5.7. Safety Parameter

No clinical relevant safety issue was detected during the investigational period.

## 6. Discussion

Multiple studies have been already published postulating the beneficial cellular effects of oral curcumin [1–3]. The recently published article by Chuengsamarn et al. reported a benefit of daily oral doses of 1.5 g of curcumin capsules, lowering the number of incidences of type 2 diabetes mellitus in a prediabetes population and improving overall *β*-cells functioning [24].

The underlying molecular mechanisms of curcumin are largely unknown. The HO-1 expression was thought to be one of the possible pathways of curcumin action, but in the current study we could not detect any significant increase in HO-1 mRNA and protein concentration after a single high dose of 12 g oral curcumin at any timepoint throughout a reasonable observation period of 48 hours (Figures 3(a) and 3(b)).

Heme oxygenase-1 is identified as particularly important in protection against a large number of diseases resulting from increased production or decreased removal of reactive oxygen species [25]. Relevant, for the present study, HO-1 was observed to be induced by curcumin *in vitro* in hepatocytes and *in vivo* in urinary epithelial cells [16, 26].

HO-1 expression was analyzed in PBMCs because these cells are regarded as important mediators of inflammation in a large number of diseases and are also considered to be prime targets of the cytoprotective actions of HO-1 [27]. The human specific (GT)n polymorphism of HO-1 gene has been reported to be associated with various chronic diseases [13–15, 28, 29], where in general a shorter (GT)n repeat polymorphism has protective effects and resulted *in vitro* in a higher HO-1 baseline activity and increased inducibility [10–12]. However, it has not yet been demonstrated that the (GT)n polymorphism is relevant to a pharmacological induction of HO-1 in humans. Furthermore, we could show that the (GT)n polymorphism does not influence HO-1 induction by heme arginate in healthy males in a clinically relevant way [23]. In the present study, stratifying our cohort according to the (GT)n polymorphism did not display any difference in the parameters observed.

Animal studies have shown that orally administered curcumin has low absorption rates (60–66%) and about approximately 75% of the ingested curcumin is excreted unmetabolized in the feces and only negligible amounts in the urine [30, 31]. Pharmacokinetic studies in humans have generally produced similar data [19, 21, 32]. Plasma levels of curcumin could only be measured in higher orally administered doses (8 to 12 g/day) [19]. Due to curcumin's poor systemic bioavailability after oral dosing usually piperine, a known inhibitor of hepatic and intestinal glucuronidation was used as an additive to enhance the therapeutic use of curcumin. Shoba et al. have shown that the poor bioavailability is increased by piperine, in rats by 154% and in humans by up to 2000% [22]. Despite the fact that the study drug contained 5 mg of Bioperine, no detectable curcumin plasma levels could be observed.

A recently performed clinical trial generated tolerability and clinical and biomarker efficacy data on Curcumin C3 Complex in persons with Alzheimer's disease. Due to the low measured plasma levels, no clinical or biochemical evidence of efficacy of Curcumin C3 Complex could be demonstrated [33]. However, the lack of curcumin traces in plasma after oral dosing does not support a direct curcumin-mediated effect and urges for further investigations to present the pharmacological activity and clinical mode of action of this dietary constituent.

To understand the pharmacological action of curcumin, an analysis of plasma concentrations, conjugates, and metabolites (curcuminsulfate, tetrahydrocurcumin, and hexahydrocurcumin) would be imperative for any ongoing clinical investigation. Furthermore, the molecular steps and signal transduction pathways underlying the HO-1 upregulation by curcumin remain largely unknown.

## 7. Limitation

Regulation of promoter activity by the (GT)n repeat length may differ in blood cell types and in response to different stimuli [10, 11]. We do not exclude the possibility that the proportion of HO-1 expressing cells varied across PBMCs tested in S/S and L/L groups. Further investigations are needed to determine whether different PBMCs subpopulations respond differently to curcumin. Since a single high dose of oral curcumin was investigated in the current study, no observation on altered resorption after multiple dosing was performed.

In this clinical trial we used the HPLC-MS method for the analysis of plasma curcumin concentrations since this assay was successfully applied to the pharmacokinetic studies of curcumin in rats [34]. Additional assays could provide more useful information on the determination of curcumin in human plasma samples.

## 8. Conclusion

The present study shows that despite a single high oral dose of curcumin, no measurable plasma levels could have been detected nor any change in the HO-1 expression could have been detected. The present work builds on previous demonstrations that curcumin strongly upregulates HO-1 activity, an effect that could explain one of the pharmacological actions of curcumin. It is anticipated that increasing clinical use of curcumin and other inducers of HO-1 activity will be of enormous benefit to healthcare worldwide.

## Figures and Tables

**Table 1:** Baseline characteristics.

Baseline characteristics	S/S (*n* = 5)	L/L (*n* = 5)
(GT)n repeats	23.4 ± 1.07	31.5 ± 2.92
Age (years)	29.4 ± 3.71	27 ± 3.94
Weight (kg)	81.6 ± 9.47	80.4 ± 11.19
BMI (kg/m^2^)	23.68 ± 1.98	23.68 ± 1.75
Conjugated bilirubin (mg/dL)*	0.15 ± 0.03	0.195 ± 0.08
Unconjugated bilirubin (mg/dL)	0.76 ± 0.21	0.71 ± 0.27
HO-1 mRNA (ΔCT)	4.31 ± 1.25	4.38 ± 1.15
HO-1 protein (HO-1/*β*-actin)	0.13 ± 0.15	0.13 ± 0.14

Baseline characteristics of the S/S and L/L groups. Data are presented as means ± SD. Significant statistical differences are reported in the text (*P = 0.015). (GT)n repeats: GT length polymorphism; BMI: body mass index; HO-1 mRNA (ΔCT): HO-1 mRNA level as cycle threshold difference compared to the reference gene (18S).

## Abbreviations

AUC: Area under the curve
CO: Carbon monoxide
EOS: End of study
(GT)n: Dinucleotide guanosine thymine repeat
HO: Heme oxygenase
L/L: Homozygous for the long GT repeat length polymorphism in the promoter of the HO-1 gene
PBMCs: Peripheral blood mononuclear cells
S/S: Homozygous for the short GT repeat length polymorphism in the promoter of the HO-1 gene.
